# Assessment of 24 h Sodium and Potassium Urinary Excretion in Normotensive and Hypertensive Dominican Adults

**DOI:** 10.3390/nu15143197

**Published:** 2023-07-19

**Authors:** Carlos Heriberto García-Lithgow, Madeline Durán-Cabral, Alexandra Winter-Matos, Kilsaris García-Estrella, Julen García-Durán, Estefanía Di-Sanzo, Nicole Martínez-De-La-Cruz, Julia Rodríguez-Abreu, Begoña Olmedilla-Alonso

**Affiliations:** 1 Centro Cardio-Neuro-Oftalmológico y Transplante (CECANOT), Santo Domingo 10306, Dominican Republic; cgarcial@unphu.edu.do (C.H.G.-L.); alexandra.winter@cecanot.com.do (A.W.-M.); kilsarys.garcia@cecanot.com.do (K.G.-E.); estefania04@gmail.com (E.D.-S.); nicolemaryce@gmail.com (N.M.-D.-L.-C.); 2 Centro de Diagnóstico, Medicina Avanzada y Telemedicina (CEDIMAT), Santo Domingo 10216, Dominican Republic; julenalejandro@gmail.com (J.G.-D.); jjrodriguez@cedimat.net (J.R.-A.); 3 Facultad de Ciencias de la Salud, Universidad Nacional Pedro Henríquez Ureña (UNPHU), Santo Domingo 10602, Dominican Republic; 4 Dirección de Investigación, Universidad Nacional Pedro Henríquez Ureña (UNPHU), Santo Domingo 10602, Dominican Republic; 5 Departamento de Metabolismo y Nutrición, Instituto de Ciencia y Tecnología de Alimentos y Nutrición (ICTAN-CSIC), 28040 Madrid, Spain

**Keywords:** Dominicans, hypertensive, salt intake, sodium and potassium excretion, sodium-to-potassium ratio

## Abstract

Higher salt (sodium) intake has been associated with higher blood pressure (BP). The degree of association may be influenced by factors such as age, origin, and dietary components. This study aimed to evaluate the 24 h urinary sodium (Na) and potassium (K) excretion in normotensive and hypertensive Dominican adults and estimate their salt intake. 163 volunteers (18–80 years old) participated in a cross-sectional study. The 24 h Na and K urinary excretion were measured using an ion-selective electrode technique. Na and K urinary excretion (99.4 ± 46.5 and 35.0 ± 17.5 mmol/24 h) did not correlate with BP, except in the normotensive group, in which K correlated with SBP (0.249, *p* = 0.019). Na and K excretion were similar in normotensive and hypertensive subjects. When considering two age groups (18–45, 46–80 years), the Na-to-K molar ratio (3.1 ± 1.3) was higher in younger subjects (*p* = 0.040). Na-to-K ratio was associated with DBP in the total group (r = 0.153, *p* = 0.052), in the hypertensive group (r = 0.395, *p* < 0.001), and in the older group with SBP (0.350, *p* = 0.002) and DBP (0.373, *p* < 0.001). In the older group, Na-to-K ratio and DBP correlated after controlling for subjects with hypertension controlled by treatment (r = 0.236, *p* = 0.041). The Na-to-K ratio correlated, when salt intake was over 5 g/day (52.2%), with SBP (rho = 0.219, *p* = 0.044) and DBP (rho = 0.259, *p* = 0.017). Determinants of BP in the total sample were age (SBP, beta: 0.6 ± 0.1, *p* < 0.001; DBP, beta: 0.2 ± 0.1, *p* < 0.002), sex (SBP, beta: 11.2 ± 3.5, *p =* 0.001), body mass index (BMI) (SBP, beta: 1.0 ± 0.3, *p* < 0.001; DBP, beta: 0.4 ± 0.2, *p* = 0.01), and Na-to-K ratio (SBP, beta: 3.0 ± 1.1, *p* = 0.008; DBP, beta: −12.3 ± 4.0, *p* = 0.002). Sex and BMI were determinants in the younger group. Na-to-K molar ratio was determinant in the older group (SBP, beta: 6.7 ± 2.4, *p =* 0.005; DBP, beta: 3.8 ± 1.1, *p* < 0.001). The mean Na and salt intakes (2.3 and 5.8 g/day) were slightly higher and the K intake lower (1.4 g/day) than WHO recommendations.

## 1. Introduction

A large number of studies have been conducted on the association between sodium intake and cardiovascular morbidity–mortality [1,2,3], and, among other factors, a higher blood pressure (BP) has been associated with higher salt (sodium) intake [4,5] and ultimately with an increase in cardiovascular disease (CVD). However, although salt (sodium) intake is a determinant and modifiable factor of hypertension, the degree of correlation observed between the studies is inconsistent [1,3]. This could be due to the influence of other factors such as age, origin, and other dietary components [6]. Some dietary components, such as potassium, can modify the effects of sodium intake, as shown in the Dietary Approach to Stop Hypertension (DASH) study in the USA. That study demonstrated how a diet rich in potassium and calcium, together with different degrees of sodium intake (low, medium, and high), correlated positively with lower BP [7]. Studies in recent years have focused on the excretion of potassium and sodium in urine after 24 h given that the sodium-potassium correlation in the diet would appear to be more important than strictly looking at the intake of sodium in terms of its effect on BP and resulting morbidity–mortality and on CVD [6,8,9,10,11,12,13,14], proposing urinary sodium and potassium excretion as surrogate measurements of their dietary intakes as an independent predictor of stroke [15,16].

The World Health Organization (WHO) recommends reducing sodium intake to less than 2 g/day (equivalent to 5 g of salt) and increasing potassium intake (at least 3.5 g/day) to reduce BP and the risk of CVD, heart attack, and coronary heart disease among adults, with or without hypertension [17]. While these recommendations do not stipulate the optimum sodium-to-potassium (Na-to-K) ratio, individual recommendations for these two elements lead to a ratio below 1. Although the scientific community generally recommends reducing salt/sodium intake for all populations, there are discrepancies in terms of the recommended amount [18,19,20,21,22,23]. The Dietary Guidelines for Americans (DGA) recommend no more than 2.3 g of sodium/day (5.75 g salt/day) [22], while the American Heart Association (AHA) advocates a limit of less than 1.5 g of sodium/day (3.75 g salt/day), especially for those with high BP [24]. There is also disagreement regarding the impact of sodium on CVD, as emerging evidence has shown that low sodium intake poses a greater risk for certain subgroups, such as those with type 2 diabetes [25]. There are also differences in the degree of correlation between sodium and potassium intake and BP, the latter being higher in the case of hypertensive individuals with high salt intake and older adults [8].

It is estimated that the vast majority of the population consumes between 9 and 12 g of salt/day, i.e., approximately double the maximum recommended intake [21]. However, real intake could be even higher, as many data points have been obtained through dietary surveys, which, due to their degree of variability, tend to underestimate true intake [25,26]. This variability is as much due to the dietary survey methodology employed (e.g., dietary notetaking, 24 h recall, food frequency questionnaire) as to the food composition tables/databases used. Hence, whenever possible, it is more reliable to base assessments on sodium excreted in urine after 24 h, the method currently considered the “gold standard” [3,23,27,28].

Salt intake in the region included within the Pan American Health Organization (PAHO) is estimated to be above 5 g (2 g sodium) per day, varying between 8.5 and 15 g salt/person/day [29]. The PAHO therefore recommends reducing said consumption by 30% by 2025 in an attempt to prevent hypertension and CVD, the main causes of death in the region [29]. There is currently no data on salt intake in the Dominican Republic, where recent studies have revealed a prevalence of hypertension of 32.3% [30]. To define factors impacting non-transmissible disease among adults, the PAHO/WHO planned to conduct surveys in several countries, including the Dominican Republic, by 2019 [31]. However, to the best of our knowledge, there are still no data on the salt intake among Dominicans. Our aims are to estimate the salt intake by means of the 24 h urinary sodium excretion in a group of normotensive and hypertensive Dominican adults and to examine the association of urinary sodium and potassium excretion and the sodium-to-potassium ratio with BP.

## 2. Materials and Methods

### 2.1. Subjects and Study Design

A total of 165 participants (18–80 years) were selected among patients who were contacted through the cardiology and internal medicine outpatient services of two hospitals in Santo Domingo (Dominican Republic), the Centro de Diagnóstico, Medicina Avanzada y Telemedicina (CEDIMAT) and the Centro Cardio-Neuro-Oftalmológico y Transplante (CECANOT), health care personnel and administrative staff, and their relatives and acquaintances who expressed interest in participating in this study. Inclusion criteria were age > 18 years and BP: normotensive and hypertension. Volunteers were asked to report information on the following exclusion criteria: diabetes or nephropathy with complications (e.g., renal, ocular), chronic diseases, pregnancy, and consumption of restricted diets or avoidance of any food group. Two participants were excluded, one because of very high triglyceride and microalbuminuria concentrations and, the other due to a lack of analytical blood and urine data. Thus, 163 participants (78 men and 85 women) took part in a cross-sectional study (October-November 2021): 88 normotensive and 75 hypertensive subjects. Classification as normotensive or hypertensive was conducted using systolic blood pressure (SBP) ≤ 140 mm Hg and/or diastolic blood pressure (DBP) ≤ 90 mm Hg as the cut-off point [32,33,34], in line with the criteria established by the European Society of Cardiology (ESC) and European Society of Hypertension (ESH) in their clinical practice guidelines [34].

The volunteers included in the study underwent fasted blood and 24 h urine sampling and blood pressure and anthropometric measurements. Three-day 24 h dietary recalls were used for dietary assessment and will be published elsewhere along with two questionnaires, a general one about global health and dietary habits based on everyday food consumption in Dominican homes (the family food basket and a PAHO survey on knowledge, attitude, and use of salt in food) [35].

The study was conducted in accordance with the guidelines laid down in the Declaration of Helsinki, and all procedures involving human subjects were approved by the Consejo Nacional de Bioética en Salud (CONABIOS) (registry No. 022-2020, dated 9 December 2020). In the Dominican Republic, participant identity was preserved during the handling of samples and data in accordance with Law No. 172-13, G.O., and No. 10737 of 15 December 2013. Written informed consent was obtained from all subjects.

#### 2.1.1. Anthropometric and Blood Pressure Measurements

Anthropometrical measurements included height, weight, waist and hip circumference, and bioimpedance analysis (Omrom HBF-514C-LA). Body weight (kg) was measured without shoes and with light clothing. Height was recorded to the nearest cm. using a scale (8023 Jiangsu Medical SH, Jiangsu Scale). Body mass index (BMI) was calculated as weight (kg)/height (m^2^).

BP (mm Hg) was taken using an automatic BP monitor (Omrom Hem-7320-LA) in quiet and temperature-controlled conditions. The cuff was placed on the right upper arm. BP was measured three times, with at least five minutes between measurements.

#### 2.1.2. Urine and Blood Samples

The 24 h urine samples were stored in two-liter plastic containers with 10 g of boric acid as a preservative before transfer to the laboratory. Sodium, potassium, creatinine, microalbumin, and endogenous creatinine clearance (ECC) were analyzed and ECC was corrected by body surface area (ECC-c). A 24 h sodium and potassium urinary excretion was analyzed using indirect potentiometry with the membrane ion-selective electrode technique using the AU5811 Beckman Coulter analyzer (Beckman Coulter Inc., Brea, CA, USA).

A fasted blood sample was obtained to analyze the lipid profile (cholesterol, HDL- and LDL-cholesterol, VLDL, triglycerides), creatinine, and glycemia. Cholesterol was analyzed based on cholesterol dehydrogenase, HDL-cholesterol using direct polymer-polyanion measurement, triglyceride using an enzymatic method, and microalbumin with an immunoturbidimetric test. Creatinine was analyzed using the kinetic alkaline picrate method (a modification of the Jaffe procedure kinetic method) and glucose by the hexokinase method. Body surface area [36], endogenous creatinine clearance (ECC) and ECC-corrected by surface area (ECC-c) were calculated using the following formulas:

ECC = Creatinine in urine (mg/dayL) × diuresis (mL urine 24 h)/creatinine in serum (mg/dayL) × 1440

ECC-c = Creatinine in urine (mg/dayL) × diuresis (mL urine 24 h) × 1.73/creatinine in serum (mg/dayL) × 1440 × body surface area

Body surface area = √ (weight (kg) × height (cm))/3600

To validate 24 h urine collection, a correlation between fat-free mass calculated from data obtained by electrical bioimpedance and that determined via urinary creatinine excretion was calculated. The fat-free mass was calculated using the following formula (cited in [37]):

Fat-free mass (kg) = 7.38 + 0.02908 × urinary creatinine (mg/day)

All the analytical determinations in urine and blood were performed using an AU5821 Beckman Coulter analyzer (Beckman Coulter Inc., Brea, CA, USA) in the Amadita P. de González S.A.S. Clinical Laboratory, which implemented and maintained a Quality Management System (ISO 9001:2015) [38].

#### 2.1.3. Statistical Analysis

To assess differences between normotensive and hypertensive subjects, the sample size was calculated on the basis of the urine sodium excretion data (140.5 ± 34.6 y 150.4 ± 38.8 mEq/d, respectively) [39] using the G*Power Program (Universität Düsseldorf, www.gpower.hhu.de/ accessed on 02/03/2021). A sample size of 77 subjects per group was necessary to obtain a difference in urine sodium excretion (10 mEq/d) with 80% power and an alpha error of 0.05.

The data are expressed as the mean, standard deviation, and median. The normal data distribution of the data was assessed (Kolmogorov-Smirnov test). Correlations among urine and serum variables, blood pressure, and anthropometric measurements in the total sample and in the subgroup of participants with a salt intake higher than 5 g/day (*n* = 85) were established using Spearman’s rho correlation coefficient. All reported *p*-values are based on a two-sided test, and a *p* value < 0.05 was considered to indicate statistical significance. IBM^®^ SPSS^®^ Statistics for Windows, version 27.0, was used for all statistical calculations.

Partial correlation matrices for each age group controlled by BMI (3 categories: normal weight (18.5–25 kg/m^2^), overweight (25.1–30 kg/m^2^), and obesity (≥30.1 kg/m^2^) assessed the correlation between SBP and DBP with all other variables.

In the total sample (age as a continuous variable) and in each age group (18–45 and 46–80 years), the relationship between 24 h sodium and potassium excretion in urine and BP (SBP and DBP as dependent variables) was subjected to linear mixed model regression analysis, considering sex and BMI (normal weight, overweight, and obesity) as fix factors and urinary sodium, potassium, Na-to-K ratio, sodium-to-creatinine ratio, and potassium-to-creatinine ratio as covariates.

A linear model was used to assess DBP and SBP correlations, considering sex and BMI as fixed factors and the Na-to-K ratio as a covariate (in urine). The correlation matrix to assess the R value in the model was calculated from the correlation matrices of the original values of the variables and the values predetermined by the model.

## 3. Results

**Figure 1:**
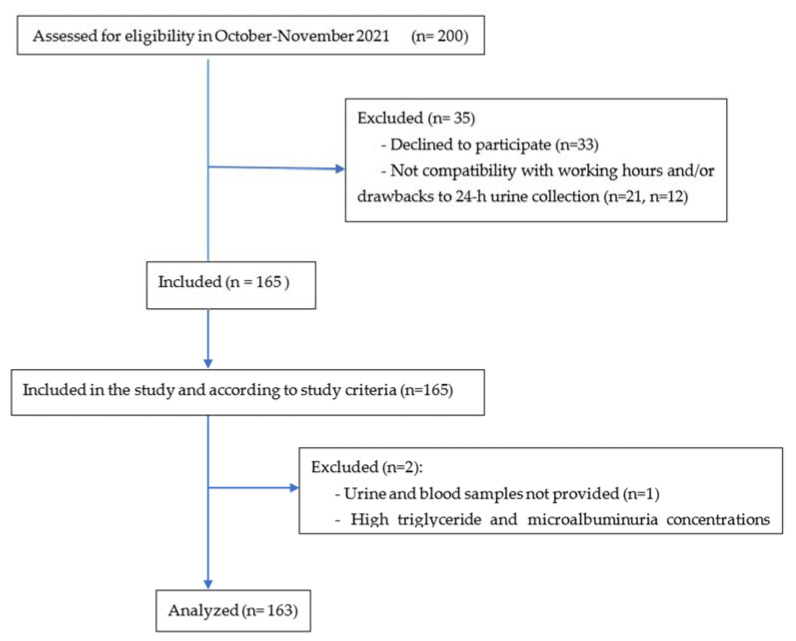
Flow diagram for study participants.

Participant characteristics are shown in Table 1, and a flow diagram for study participants is shown in Figure 1. They were recruited at the hospitals CEDIMAT (*n* = 85, 52.1%) and CECANOT (*n* = 78, 47.9%), with 78 men (47.9%) and 85 women (52.1%). The average age was 44.5 ± 14.6 y (age range: 18–80 y). By racial phenotype, the group was 76.7% mixed race (mulatto), 12.9% black, 9.2% white, and 1.2% Asian. A similar number of hypertensive (46%) and normotensive (54%) subjects were included in the study. Most (82.7%) of the hypertensive group (*n* = 75) were aware of their condition, with 62 of them being treated for hypertension (most frequently used: Amlodipino (19), Losartan (16), Candesartan (13), Valsartan (8), Bisoprolol (7), or Enalapril (5)). Of these participants treated with antihypertensives, only 34 had their hypertension controlled. Only 12% of the participants were diabetics.

Table 2 shows data on BP, anthropometric measurements, and biochemical data in 24 h urine (sodium, potassium, creatinine, and ratios) and blood (lipid profile, creatinine and glycaemia) in the total sample as a whole and grouped by BP (normotensive and hypertensive). The urine sample was appropriate according to validation by means of the correlation between muscle mass and urinary creatinine (r = 0.717).

SBP and DBP were higher in hypertensive than in normotensive subjects, as were the BMI (*p* < 0.001), the waist-to-hip ratio, body fat, and visceral fat (*p* < 0.001). Muscle mass was lower than in the normotensive group (*p* = 0.038). The 24 h urine sample showed no difference for sodium or creatinine, but significant differences were found for ECC-c (within the reference ranges in all groups), higher in normotensive (*p* = 0.041), and microalbumin (higher in hypertensive, *p* = 0.005). The sodium intake, but not the potassium intake, correlated significantly with the ECC in the total sample (Na-intake and ECC: rho = 0.179, *p* = 0.023). On comparing the age groups, those correlations were observed in the older group (Na-intake and ECC: rho = 0.289, *p* = 010; K-intake and ECC: rho = 0.246, *p* = 0.029). Blood tests showed similar total cholesterol for both groups, but HDL-cholesterol was higher in the normotensive group (*p* = 0.002), and VLDL-cholesterol and triglycerides were higher in the hypertensive group (*p* = 0.006 and *p* = 0.004, respectively), as was the glycemia (*p* = 0.01).

Urinary excretion of sodium, potassium, and creatinine was similar between normotensive and hypertensive subjects. To validate 24 h urine collection, a correlation was drawn between fat-free mass calculated from data obtained by electrical bioimpedance (51.2 ± 12.5 kg) and that determined via urinary creatinine excretion (48.7 ± 16.6 kg), and a significant positive correlation was found (rho = 0.717, *p* < 0.001).

The age of participants ranged from 18 to 80 years, and two age groups [18–45 y (51.5%) and 46–80 years (48.5%)] were established given that age was a confounding factor. The cut-off point for the age was set at 45 years based on other studies of representative populations [5,40,41] and because 44% of Dominican adults in the age range of 40–49 years have been described as hypertensive [42]. BP data, anthropometric measurements, and biochemical data in 24 h urine and blood samples of participants grouped by age are shown in Table 2. Significant differences were found in BP and anthropometric and blood data between young and older subjects, similar to those obtained from the comparison between normo- and hypertensive subjects. However, comparisons between the two age groups showed more statistical differences in 24 h urine and creatinine excretion (higher in the younger group, *p* < 0.001), ECC and ECC-c (higher in younger subjects, *p* < 0.001), the Na-to-K ratio (higher in younger subjects, *p* = 0.04), and the K-to-creatinine ratio (higher in older subjects, *p* = 0.013). However, there was no difference in the excretion of sodium or potassium or in the sodium-to-creatinine ratio.

Table 3 shows correlations between BP and variables in urine, blood, and anthropometric measurements in the sample as a whole, between the two age groups, and between normotensive and hypertensive subjects. Sodium and potassium excretion did not show any correlation with BP except in normotensive subjects, with a significant correlation between potassium and SBP. The Na-to-K ratio was found to be significantly correlated with DBP in the total group, in the older group with SBP and DBP, and in the hypertensive subjects. In the older group, the sodium-to-creatinine ratio was correlated with SBP. Anthropometric measurements, creatinine in serum, and ECC-c showed several significant correlations in the group as a whole and in the age groups.

In a subgroup of participants with a salt intake higher than 5 g/day (*n* = 85), the Na-to-K ratio showed a significant correlation with the SBP (rho = 0.219, *p* = 0.044) and DBP (rho = 0.259, *p* = 0.017).

In the partial correlation matrix adjusted for blood pressure (normo and hypertensive groups), the correlation between Na-to-K ratio and DBP was significantly different (r = 0.166, *p* = 0.034).

The correlation between urinary excretion parameters and BP adjusted for controlled hypertension using antihypertensive treatment showed no significant differences in the total group or in the younger group. Instead, in the older group, there was a significant correlation between the Na-to-K ratio and DBP (r = 0.236, *p* = 0.041).

In partial correlation matrices adjusted by BMI for each age group, the variable most closely correlating to BP (systolic and diastolic) was the Na-to-K ratio, especially in the older group. In the same group, SBP also correlates significantly with the sodium-to-creatinine ratio, although this effect later disappears in the analysis using the general linear model for each age group.

BMI correlated with creatinine (rho = 0.212, *p* = 0.007) and ECC (rho = 0.165, *p* = 0.035) in urine. Assessment of these correlations by age group shows that in the younger group, BMI correlated with creatinine in urine (0.357, *p* < 0.001), while in the older group, BMI correlated with ECC (0.260, *p* = 0.021) and with sodium in urine (0.264, *p* = 0.019). In the older group, sodium urinary excretion was correlated with waist size (0.397, *p* < 0.001), waist-hip size (0.379, *p* < 0.001), visceral fat (0.374, *p* < 0.001), and BMI (0.264, *p* = 0.19).

A linear model was used for each age group to evaluate the predictive value of the BP variables analyzed. The variables in the final model were sex, BMI, and Na-to-K ratio (Table 4). The determinants of SBP and DBP were different for the two age groups. In the 18–45 group, sex and BMI showed significant effects on SBP, and BMI impacted DBP. SBP was higher in men than in women (14 mm Hg) and lower in normal weight (12.1 mm Hg) than in obese subjects, and lower in overweighted (10.7 mm Hg) than in obese subjects. DBP was lower in subjects of normal weight (9.4 mm Hg) than in obese subjects and lower in overweight subjects (5.1 mm Hg) than in obese subjects. In the 46–80 group, SBP and DBP were affected by the Na-to-K ratio, and for each unit increased, SBP increased by 6.7 mm Hg and DBP increased by 3.8 mm Hg.

The R coefficients for SBP were R= 0.575 (*p* = 0.000) and R = 0.352 (*p* = 0.001) for the younger and older groups, respectively. The R coefficients for the DBP were 0.377 (*p* = 0.000) and R = 0.392 (*p* = 0.000) for the younger and older groups, respectively.

**Figure 2:**
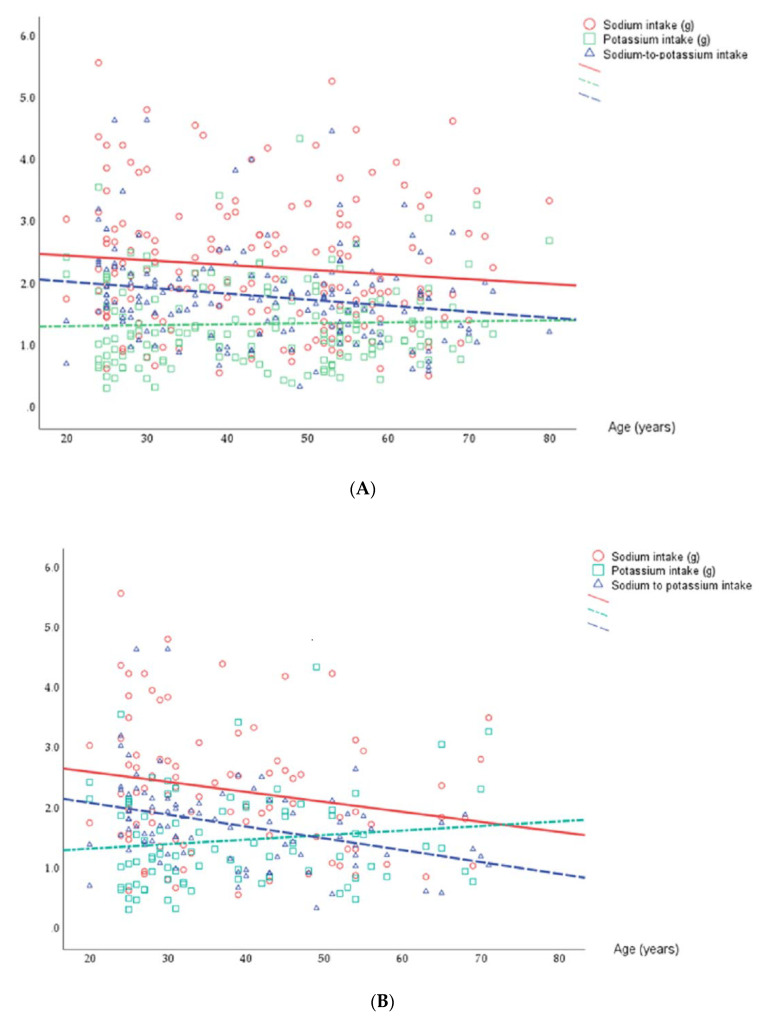

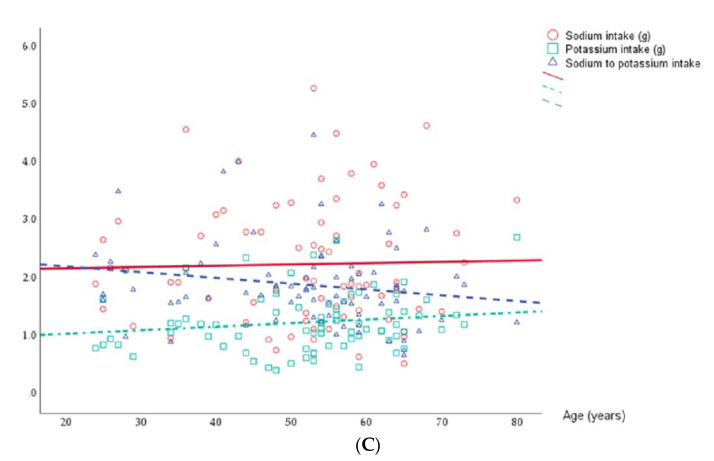
Correlation between age (years) and sodium and potassium intake (g/day) in the total sample (**A**) and in the normotensive (**B**) (*n* = 88) and hypertensive (**C**) (*n* = 75) groups.

Table 5 and Figure 2 show the salt, sodium, and potassium intakes estimated from the 24 h sodium and potassium urinary excretion for the entire sample and age and BP groupings. The mmol 24 h sodium and potassium urinary excretion were converted to g/day (0.023 mgNa^+^  =  1 mmol Na^+^ or 1 mEq Na^+^; 0.039 mgK^+^  =  1 mmol K^+^ or 1 mEq K^+^). The conversion from sodium to sodium chloride (or dietary salt) by multiplying by 2.54. There was no significant difference between normo- and hypertensive subjects in terms of sodium intake, but there was a significant difference in terms of potassium intake in the group as a whole and in the older sub-group, with potassium intake being higher in the normotensive subjects. In the older group, normotensive subjects exhibited lower sodium and higher potassium intake, leading to a lower Na-to-K ratio (*p* = 0.011) than their hypertensive counterparts.

Assuming that all of the sodium eliminated in urine (89 mmol/24 h, median value for the entire sample) (Table 2) comes from dietary intake, this excretion indicates a dietary salt intake of 5.8 ± 2.7 g (median: 5.2 g/day). Salt intake is not significantly correlated with BP but is correlated with the amount of sodium excreted in urine (rho = 1), potassium (rho = 0.652, *p* < 0.001), and the Na-to-K ratio (rho = 0.412, *p* < 0.001).

In subjects whose dietary salt intake was over the 5 g/day recommended by the WHO [17] (*n* = 85, 52.2%), but not in those consuming less, the Na-to-K ratio correlated significantly with SBP (rho = 0.219, *p* = 0.044) and DBP (rho = 0.259, *p* = 0.017).

## 4. Discussion

To the best of our knowledge, there is no data on the salt intake of Dominicans, a population with a prevalence of hypertension of 32.3% and obesity of 33.6% [30], and this is the first study on salt intake in a group of Dominican adults, assessed by means of the “gold standard” method, the 24 h sodium excretion in urine [3,27,28]. A similar number of adults, women and men, participated (52 vs. 48%, respectively), with a mean age of 44.5 (± 14.6) y. Most participants were mulatto (77%) and, in a smaller proportion, black or white (13% and 9%, respectively), representative of the Dominican population [43]. Most of the participants were non-diabetics (88%), a percentage in line with diabetes data in Dominicans [30,44]. Only 17% of the participants were unaware that they had high BP, a small percentage compared with the 40.6% who did not know about their high BP, according to a recent survey from the Dominican Government [30].

### 4.1. Urinary Sodium and Potassium Excretion

The median 24 h urinary sodium excretion in the total sample of 2046 mg/day (mean 2286 mg/day) was in the lower range of the 24 h sodium excretion reported in a systematic review that included more than 10,000 adults from the US and Europe (median: 3270 mg/24 h, 10th to 90th percentile, 2099 to 4899) [3]. Urinary sodium excretion in our study was also below the lower interquartile reported for a representative sample of non-hypertensive Spanish adults (median: 2613, interquartile: 2480–4942 mg/day; mean: 3062 mg/day) [26]. This sodium excretion was just half of the sodium excreted in a representative sample of the adult Irish population (4078 mg/day) [5] and in a healthy adult sample in northern Greece (4220 mg/day) [45] and was lower than the mean reported for men and women in Norway (3535 mg/day) [40], in the US (3291 mg/day by 24 h dietary recall and mean: 3608 mg/day, median: 3320 mg/day, by 24 h urinary sodium excretion) [14,41], and in Greece university students (2803 mg/day) [12]. Urinary sodium excretion in this study was much lower than in China (4300 mg/day and 4700 mg/day) [16,46], in 18 countries from America (north and south), Asia, Africa, and Europe (42% from China), 4930 mg/day [8], and Kazakhstan (6782 mg/day) [47].

Surprisingly, the median 24 h potassium excretion of 1209 mg/day (mean: 1365 mg/day) in this study was substantially lower than the mean reported in the previously mentioned data from the US and Europe (2535 mg/day, median range: 2067–3610) [3] and lower than the median reported for men and women in Spain (2613 mg/day) [26] and in Ireland (3290 mg/day) [5], and lower than the mean reported in Norway (3420 mg/day [40], in the US (2542 mg/day by 24 h dietary recall and 2155 mg/day by 24 h urinary sodium excretion) [14,41], in Greece (2152 mg/day and 3303 mg/day) [12,45], in Kazakhstan (2271 mg/day) [47], and in 18 countries from America (north and south), Asia, Africa, and Europe (42% from China) 2120 mg/day [8]. Potassium excretion was similar to that in the lower range of the distribution in the Chinese population (mean: 1600 mg/day, range: 1160–1880) [46] and lower in hypertensive Chinese adults (*n* = 189 males; 2100 mg/day) [16].

Low 24 h sodium and potassium urinary excretion was observed in the overall sample, and no difference was found in terms of the age of the subjects, coinciding with findings from other studies [41], despite the fact that age could contribute to an increase in urinary sodium and potassium losses due to a decline in the glomerular filtration rate and an increased incidence of renal disease with advancing age. Lower potassium urinary concentration has been described in younger vs. older subjects [26] and higher sodium excretion in younger subjects [48] and the opposite, higher sodium excretion in older subjects [49]. Race has been associated with potassium excretion and intake, both of which are lower in blacks than in whites [6]. Therefore, this variable may have been important in the results of this study considering the high percentage (77%) of mulatto subjects. Also, the very low K excretion could be related to lower consumption of potassium-rich foods, and, as reported in a group of Dominicans, the consumption of fruit and vegetables (major potassium food sources) was below the recommendations for Dominicans [50]. Moreover, only a very limited variety of fruit and vegetables are most frequently consumed by Dominicans, as can be deduced from a recent survey on the food intake in this country [51]. In addition to dietary factors, sweat and fecal losses of these compounds [52] should be taken into account for a more accurate estimate of their excretion. In sweat, the sodium losses have been estimated at approximately 10% under normal conditions [53] and higher depending on the ambient temperature [54]. Instead, the potassium losses stay relatively constant, regardless of sweat rate and level of acclimatization [55]. Thus, this sodium loss could be greater in countries with a tropical climate such as the Dominican Republic (during the study, the mean temperature was 32 °C and the relative humidity was 86%), which could partially account for the lower sodium and potassium urinary excretion observed in this study compared to people from other countries.

Sodium excretion was similar in normotensive and hypertensive subjects, which contrasts with the lower excretion in normotensive subjects described by others [38,49]. Potassium excretion was also similar in normotensive and hypertensive patients, which does not coincide with the inverse association between higher potassium intake (based on 24 h dietary recall) and BP at a higher threshold in a USA survey (NHANES 2018) [14]. As anticipated decades ago, potassium intake may be a major factor in the epidemiology of hypertension [6].

The Na-to-K ratio was approximately three, and there was no difference in terms of BP levels, but there was a difference according to age, with the ratio being higher in the younger group as well as the potassium-to-creatinine ratio, creatinine, ECC, and ECC-c. This molar ratio is far from the recommended value of less than 1 (based on the sodium and potassium intake recommendations by the WHO [21]), but is similar to the ratios described in cross-sectional studies of adults (from 2.2 to 3.8), with a Na-to-K ratio greater than 2 [5,41,45] and lower than that found in other populations such as Kazakhstan (6.34) [47].

BP was higher in the older group, as has also been described in other studies (i.e., [38]). Considering age and BP, the older normotensive subjects exhibited lower sodium intake and a higher potassium intake than the older hypertensive group, which led to a lower Na-to-K ratio intake (=1.22) and closer to the target of 0.6 (based on Na and K intakes recommended by the WHO) [21]. According to a recent meta-analysis on cardiovascular risk in several prospective studies controlled for confounding factors, higher sodium excretion, lower potassium excretion, and a higher Na-to-K ratio are associated with a higher cardiovascular risk [3]. Hence, the older normotensive subjects should have a lower cardiovascular risk compared with the older hypertensive group. While evidence on the effect that the Na-to-K ratio has on BP highlights the benefits of reducing sodium and increasing potassium compared to sodium and potassium separately [11], in this study, the increase in K intake (which is extremely low) seems to be more necessary than a decrease in the Na intake. However, an increase in dietary potassium intake should not be advised for those with impaired kidney function who are in an advanced state of chronic kidney disease. Therefore, an education program to help lower the sodium-to-potassium ratio and individualized approaches to that end may help to minimize the gap with recommended levels [11].

The correlation between the intake of sodium and potassium and BP varies in the literature, but, in general, this correlation is higher in hypertensive people, in persons consuming high-sodium diets, and in older persons [8,26], although no significative association has also been described [47]. In this study, BP did not correlate significantly with sodium and potassium excretion in the total sample, which coincides with the findings described in some studies [47,48], but not with the association between BP and sodium urinary excretion described by others [26,39]. Instead, the Na-to-K molar ratio correlated with SBP and DBP in the older group and with those with a high salt intake (>5 g/day). Similarly, that association has been shown in other studies [9,56].

Predictive BP variables were different depending on age group, sex, and BMI for the younger group and Na-to-K molar ratio for the older group. In the younger group, higher SBP was measured in men than in women, as also described recently in the Dominican Republic and other populations [26,40,49], and both SBP and DBP increased with body weight. This association between body weight and BP has been widely observed (i.e., [26]), and weight loss in overweight and obese individuals is one of the non-pharmacological interventions recommended for US adults with high BP [34].

In the older group, only the Na-to-K ratio was a determinant of SBP and DBP, and for each unit increase in this ratio, there was an increase of 6.7 mm Hg in SBP and 3.8 mm Hg in DBP. Similar BP increments associated with the Na-to-K ratio have been reported in a study compiling data from eighteen countries (42% from China) [8]. Thus, the Na-to-K ratio seems to be a more important predictor of hypertension than either sodium or potassium intake alone, in agreement with other authors [3,5,9,11,14,16,41] in the older group.

#### 4.1.1. Estimated Sodium, Salt, and Potassium Intake: Comparison with Recommended Intake

The sodium 24 h urinary excretion (median in the entire sample: 89 mmol/24 h = 2046 mg/24 h) corresponded to a dietary salt intake of 5.8 g (median: 5.2 g, range: 1.34 to 14.2 g salt/day), which is quite a bit lower than the intake estimated from 10 countries in the PAHO region (8.5–15 g/day, data from 2015) [29]. Our result is around half of the salt intake data obtained by 24 h sodium urinary excretion in Mesoamerica (3.5 and 3.8 g/day, equivalent to 8.9–9.8 g salt/day) [48,57]. This discrepancy between the estimated salt intake at a population level in the Region and our data in a group of Dominicans could be due to the origin/race of the population and their culinary habits, the salt intake assessment method, and also the fact that a single 24 h urine collection may not reliably reflect an individual’s usual intake [23]. In Spain, a different geographical area and climate, in a representative sample, or in northern Greece, 88.2% and 94.4%, respectively, had salt intakes above the recommended 5 g/day [26,45], a percentage considerably higher than the one found in the present study (52.2%). In the Spanish population, sodium urinary excretion was correlated with SBP and DBP, but in the present study, only the Na-to-K ratio was associated with BP in the older group.

The mean potassium intake in our study, 1.4 g/day (median 1.2 g/day), is far from the minimum of 3.5 g potassium/day (90 mmol/d) recommended by the WHO with the aim of reducing BP [21] and also by the EFSA for the adult population [55] and the 4.7 g/day suggested by the DGA and by the AHA [22,24]. These sodium and potassium dietary intake recommendations (g) lead to Na-to-K ratios of 0.32 [22], 0.5 [24], and in the range 0.6–1 [21,23,55], which are approximately three times lower than the ones obtained in this study. Our data showed almost “normal” sodium but very low potassium urine excretions; thus, a higher potassium consumption would lead to a better Na-to-K ratio and consequently to lower BP and a lower risk of stroke associated with potassium intake below 3500 mg/day [54].

The median salt intake is higher, but closer to the maximum 5 g salt/day (under 2 g sodium/d) recommended by the WHO with the aim of reducing BP [58], and to the 5 g salt/day considered by the European Food Safety Authority as a safe and adequate intake for the general European adult population [23]. This is also higher than the 3.75 g of salt/day (1.5 g of sodium/day) estimated as adequate intake for the general population by the DGA [22], but is lower than the 2.3 g of sodium/day recommended by the AHA for the general population as part of a healthy diet [24].

#### 4.1.2. Strengths and Limitations of This Study

The main strengths of this study are the use of a 24 h urine sample for the analysis of sodium and potassium, which is the “gold standard” method for assessing salt intake, and the application of a validated urine collection protocol. However, the use of only one 24 h urine collection per subject is a valid approach at a population levels [28], but it is poor at an individual level because of their day-to-day variability [40]. In addition, the sample size was relatively small and not representative of the Dominican population.

## 5. Conclusions

Although neither sodium nor potassium urinary excretion were associated with BP, except the potassium in the normotensive group, the Na-to-K molar ratio (around 3) was associated with blood pressure in the older subjects (age 46–80), in hypertensive subjects, and in subjects with a salt intake above 5 g/day. This Na-to-K ratio was lowest for the older normotensive group due to their relatively higher potassium intake.

There were no differences in the salt intake estimated in the normotensive and hypertensive Dominican subjects in this study, which, on average, was slightly higher (5.8 g salt/day) than that recommended by WHO (5 g/day) [21,58]. Potassium intake was higher in normotensive subjects and substantially lower than recommended (3.5–4.7 g/day, [21,59]).

BP determinants were age, sex, and BMI in the total sample and sex and BMI in the younger group. The Na-to-K ratio was a BP determinant in the older group. This study, with the first data set on the salt intake in adult Dominicans, may serve as the basis for a further study in a representative sample of the Dominican population and to define public health strategies based on age.

## Figures and Tables

**Table 1:** Personal and anthropometric characteristics of the participants (*n* = 163).

Age (Years)	44.5 ± 14.6 (18–80)
Sex	Men 78/women 85
Race	Mix-race (mulatto) 125/Black 21/White 15/Asian 2
Diabetes	No 143 (87.7%)/yes 20 (12.3%)
Normotensive/Hypertensive	88 [56 mulatto, 16 black, 3 white]/75 [69 mulatto, 4 black, 13 white/2 Asian]

**Table 2:** Blood pressure and biochemical data in 24 h urine and blood. Mean ± SD [median].

	Total Sample (*n* = 163) 78 M, 85 W	Normotensive (*n* = 88) 43 M, 45 W	Hypertensive (*n* = 75) 35 M, 40 W	*p* Value	18–45 Years (*n* = 84) (Normotensive = 64, M = 37, W = 27) (Hypertensive = 20, M = 11, W = 9)	46–80 Years (*n* = 79) (Normotensive = 24, M = 6, W = 18) (Hypertensive = 55, M = 24, W = 31)	*p* Value
Systolic blood pressure (mm Hg)	126.2 ± 21.1 [123.3]	116.3 ± 11.8 [115.8]	138.0 ± 23.4 [135]	<0.001	119.3 ± 15.5 [120.2]	133.7 ± 23.6 [128.6]	<0.001
Diastolic blood pressure (mm Hg)	81.2 ± 11.2 [81.0]	76.6 ± 7.3 [76.6]	86.7 ± 12.5 [87.3]	<0.001	78.7 ± 10.6 [77.6]	84.0 ± 11.2 [83.6]	0.001
Heart rate (bpm)	71.3 ± 10.2 [71.6]	71.0 ± 9.8 [70.8]	71.5 ± 10.8 [72.0]	0.826	72.2 ±10.7 [73.0]	70.2 ± 9.7 [69.3]	0.077
Anthropometric measurementes							
Height (cm)	166.3 ± 10.0 [165.5]	167.8 ± 10.6 [166.2]	164.6 ± 9.1 [164.1]	0.072	169.2 ± 10.3 [169.0]	163.2 ± 8.8 [162.0]	<0.001
Weight (kg)	80.6 ± 16.0 [79.7]	79.0 ± 17.6 [77.8]	82.4 ± 13.9 [83.1]	0.054	81.8 ± 18.1 [79.1]	79.4 ± 13.4 [80.0]	0.717
BMI (kg/m^2^)	29.1 ± 4.8 [28.6]	27.9 ± 4.7 [27.1]	30.5 ± 4.6 [30.4]	<0.001	28.4 ± 4.8 [27.4]	29.9 ± 4.7 [29.6]	0.031
Waist (cm)	95.9 ± 12.3 [95.0]	92.7 ± 12.0 [92.8]	99.5 ± 11.7 [99.0]	<0.001	93.0 ± 12.2 [92.8]	98.9 ± 11.8 [98.0]	<0.001
Hip (cm)	107.5 ± 9.0 [107.0]	106.3 ± 9.4 [104.0]	108.9 ± 8.5 [109.0]	0.015	106.5 ± 9.2 [104.2]	108.4 ± 8.8 [108.5]	0.081
Waist—hip ratio	0.89 ± 0.08 [0.89]	0.87 ± 0.07 [0.87]	0.91 ± 0.07 [0.92]	<0.001	0.87 ± 0.07 [0.88]	0.91 ± 0.08 [0.91]	0.002
Body fat (%)	36.3 ± 9.8 [35.4]	34.8 ± 9.6 [33.3]	38.1 ± 9.8 [40.3]	0.019	34.1 ± 9.7 [31.5]	38.7 ± 9.4 [40.0]	0.002
sdVisceral fat (%)	10.6 ± 4.2 [10.0]	9.2 ± 3.8 [9.0]	12.1 ± 4.0 [11.5]	<0.001	9.3 ± 4.0 [9.0]	11.8 ± 3.9 [11.0]	<0.001
Muscle mass (%)	28.5 ± 5.9 [28.0]	29.4 ± 6.4 [28.8]	27.4 ± 5.1 [27.6]	0.038	30.0 ± 6.2 [30.9]	26.9 ± 5.3 [25.9]	<0.001
Urine (24 h)							
Volume (mL)	1775.2 ± 819.1 [1740.0]	1709.4 ± 842.7 [1680]	1833.4 ± 777.2 [1780]	0.257	1732.0 ± 839.2 [1575.0]	1821.0 ± 800.0 [1850]	0.367
Creatinine (mmol)	12.4 ± 5.3 [11.5]	13.3 ± 5.3 [11.5]	11.5 ± 3.5 [11.5]	0.493	14.8 ± 8.3 [14.1]	12.0 ± 3.6 [11.2]	<0.001
Endogenus creatinine clearance (ECC) (mL/min)	108.7 ±35.7 [105.3]	113.5 ± 37.1 [115.3]	103.4 ± 33.4 [101.9]	0.089	118.0 ±35.8 [120.7]	98.8 ± 33.0 [97.1]	<0.001
ECC corrected by surface area (ECC-c) (mL/min)	100.9 ± 30.6 [102.3]	105.4 ± 30.1 [105.6]	96.3 ± 30.3 [96.9]	0.041	107.7 ± 30.5 [109.3]	93.6 ± 29.2 [93.1]	<0.001
Sodium (mmol)	99.4 ± 46.5 [89.0]	100.8 ± 47.3 [96.5]	97.9 ± 46.1 [84.5]	0.670	105.4 ± 46.8 [98.5]	92.9 ± 45.5 [81.0]	0.058
Potassium (mmol)	35.0 ± 17.5 [31.0]	37.8 ± 20.2 [33.2]	31.8 ± 13.1 [29.0]	0.123	35.2 ± 17.6 [31.0]	34.8 ± 17.5 [31.2]	0.976
Sodium: potassium ratio ^1^	3.1 ± 1.3 [2.9]	3.0 ± 1.3 [2.9]	3.2 ± 1.3 [3.0]	0.255	3.3 ± 1.3 [3.1]	2.9 ± 1.1 [2.9]	0.040
Na: creatinine ratio ^2^	9.1 ± 6.1 [7.4]	9.4 ± 7.0 [6.8]	8.8 ± 4.8 [7.8]	0.562	9.1 ± 6.7 [6.4]	9.1 ± 5.4 [8.0]	0.273
K: creatinine ratio ^2^	3.2 ± 2.3 [2.7]	3.5 ± 2.8 [2.7]	2.9 ± 1.5 [2.6]	0.067	3.0 ±2.3 [2.4]	3.5 ± 2.4 [3.0]	0.013
Microalbumin (mg)	10.4 ± 27.9 [4.8]	7.0 ± 16.2 [4.3]	14.4 ± 37.0 [5.5]	0.005	11.0 ±33.8 [4.5]	9.7 ± 20.0 [5.2]	0.616
Blood							
Cholesterol (mg/dayL)	193.0 ± 47.4 [189.7]	194.8 ± 48.0 [190.5]	190.6 ± 47.3 [189.6]	0.687	188.4 ± 44.5 [185.2]	198.3 ± 50.3 [198.7]	0.258
HDL-cholesterol (mg/dayL)	48.8 ± 14.6 [46.5]	51.4 ± 13.3 [49.1]	45.9 ± 15.7 [42.8]	0.002	50.6 ± 12.7 [48.7]	47.2 ± 16.5 [44.6]	0.051
LDL-chol.(mg/dayL)	122.2 ± 42.6 [42.6]	123.4 ± 43.9 [122.5]	120.7 ± 41.3 [125.5]	0.844	118.3 ± 42.8 [121.0]	126.7 ± 42.3 [127.0]	0.338
VLDL-cholesterol (mg/dayL)	21.9 ± 11.1 [19.5]	20.2 ± 10.8 [17.9]	24.0 ± 11.2 [20.6]	0.006	19.6 ± 11.2 [14.6]	24.5 ± 10.5 [22.3]	<0.001
Triglycerides (mg/dayL)	111.6 ± 60.6 [97.3]	101.0 ± 54.2 [89.3]	120.1 ± 55.8 [103.0]	0.004	98.0 ± 56.2 [73.2]	122.5 ± 52.7 [111.3]	<0.001
Creatinine (mg/dayL)	0.92 ± 0.28 [0.87]	0.88 ± 0.22 [0.85]	0.96 ± 0.34 [0.92]	0.137	0.94 ± 0.29 [0.93]	0.90 ± 0.28 [0.85]	0.267
Glucaemia (mg/dayL)	103.5 ± 31.1 [97.1]	97.5 ± 25.1 [95.8]	110.5 ± 35.9 [99.4]	0.010	95.1 ± 14.5 [93.3]	112.4 ± 40.4 [100.4]	0.001

*p* value between normotensive and hypertensive (Mann–Whitney, Wilcoxon). Bpm: beats per minute. M—man; W—women. ^1^ The sodium and potassium values used in this ratio were measured in millimoles. ^2^ The sodium, potassium, and creatinine values used in these ratios were measured in millimoles.

**Table 3:** Correlations [rho, (*p*)] between blood pressure and the anthropometric measurements; variables in urine and blood ^1^.

	Total Group (*n* = 163)		18–45 Years (*n* = 84)		46–80 Years (*n* = 79)		Normotensive (*n* = 88)		Hypertensive (*n* = 75)	
	SBP	DBP	SBP	DBP	SBP	DBP	SBP	DBP	SBP	DBP
Waist-hip index	**0.432 (<0.001)**	**0.259 (<0.001)**	**0.508 (<0.001)**	**0.316 (0.003)**	**0.249 (0.027)**	0.121 (0.286)	**0.497 (<0.001)**	**0.235 (0.027)**	0.219 (0.060)	0.149 (0.203)
BMI	**0.367 (<0.001)**	**0.261 (<0.001)**	**0.374 (<0.001)**	**0.326 (0.002)**	**0.264 (0.019)**	0.097 (0.395)	**0.407 (<0.001)**	**0.262 (0.014)**	0.112 (0.337)	0.006 (0.956)
Body fat	0.067 (0.394)	**0.196 (0.013)**	−0.183 (0.095)	0.197 (0.072)	0.124 (0.279)	0.028 (0.807)	−0.120 (0.264)	0.203 (0.058)	0.028 (0.816)	0.011 (0.924)
Muscle mass	0.013 (0.867)	−0.137 (0.082)	**0.333 (0.002)**	−0.127 (0.250)	−0.143 (0.211)	−0.016 (0.899)	**0.247 (0.021)**	−0.072 (0.507)	−0.018 (0.876)	−0.046 (0.698)
Visceral fat	**0.492 (<0.001)**	**0.293 (<0.001)**	**0.559 (<0.001)**	**0.318 (0.003)**	**0.294 (0.009)**	0.157 (0.169)	**0.544 (<0.001)**	**0.234 (0.029)**	0.221 (0.058)	0.083 (0.481)
Sodium	0.078 (0.323)	0.009 (0.910)	0.024 (0.827)	−0.031 (0.778)	0.194 (0.086)	0.111 (0.331)	0.125 (0.245)	−0.139 (0.196)	0.095 (0.418)	0.204 (0.080)
Potassium	0.022 (0.783)	−0.102 (0.193)	0.099 (0.370)	−0.037 (0.740)	−0.053 (0.643)	−0.141 (0.216)	**0.249 (0.019)**	−0.002 (0.986)	−0.063 (0.589)	−0.111 (0.344)
Sodium-to-potassium	0.093 (0.237)	0.153 (0.052)	−0.063 (0.567)	0.030 (0.788)	**0.350 (0.002)**	**0.373 (<0.001)**	−0.094 (0.383)	−0.114 (0.291)	0.215 (0.064)	**0.395 (<0.001)**
Sodium-to-creatinine	0.039 (0.618)	0.082 (0.299)	−0.187 (0.088)	0.024 (0.826)	**0.241 (0.032)**	0.142 (0.213)	−0.134 (0.214)	−0.055 (0.608)	**0.244 (0.035)**	**0.268 (0.020)**
Potassium-to-creatinine	−0.063 (0.427)	−0.063 (0.426)	−0.170 (0.123)	−0.036 (0.743)	−0.059 (0.604)	−0.160 (0.158)	−0.086 (0.428)	−0.020 (0.856)	0.046 (0.692)	−0.057 (0.629)
Creatinine 24 h-urine	0.116 (0.141)	−0.053 (0.505)	**0.429 (<0.001)**	0.040 (0.721)	−0.046 (0.688)	−0.035 (0.763)	**0.415 (<0.001)**	−0.007 (0.951)	−0.185 (0.113)	−0.106 (0.366)
Microalbumin	**0.219 (0.005)**	**0.187 (0.017)**	**0.262 (0.016)**	**0.269 (0.013)**	0.156 (0.169)	0.084 (0.460)	0.171 (0.111)	0.086 (0.426)	0.089 (0.450)	0.142 (0.224)
Creatinine (serum)	**0.326 (<0.001)**	0.045 (0.565)	**0.507 (<0.001)**	0.051 (0.646)	**0.239 (0.034)**	0.127 (0.264)	**0.430 (<0.001)**	0.047 (0.663)	0.180 (0.123)	−0.025 (0.829)
CCE-c	**−0.195 (0.013)**	**−0.185 (0.018)**	−0.087 (0.429)	−0.149 (0.175)	−0.218 (0.054)	−0.146 (0.198)	−0.006 (0.956)	−0.174 (0.104)	**−0.334 (0.003)**	−0.142 (0.223)

^1^ Significant correlations are highlighted in bold.

**Table 4:** Linear mixed model analysis of biochemical and anthropometric factors, sex, and age data associated with blood pressure (SBP and DBP).

Systolic Blood Pressure					
		beta	S.E.	*p*	95% CI
Total sample	Constant	96.7	9.9	0.000	77.3, 116.1
	Age	0.6	0.10	<0.001	0.4, 0.8
	Sex (man)	11.2	3.5	0.001	4.4, 18.1
	Sex (woman)	0			
	BMI—normoweight	−12.3	4.0	0.002	−20.2, 4.4
	BMI—overweight	−8–3	3.2	0.009	−14.5, 2.1
	BMI—obese	0			
18–45 years	Constant	114.4	4.8	0.000	105.0, 123.8
	Sex (man)	14.02	2.87	0.001	8.40, 19.64
	Sex (woman)	0			
	BMI—normoweight	−12.07	3.72	0.001	−19.35, −4.79
	BMI—overweight	−10.7	3.2	0.001	−16.95, −4.49
	BMI—obese	0			
46–80 years	Constant	118.6	7.7	0.000	103.6, 133.6
	Sodium-to-potassium ratio	6.7	2.4	0.005	2.07, 11.34
Diastolic blood pressure					
		beta	S.E.	*p*	95% IC
Total sample	Constant	70.8	5.8	0.000	59.5, 82.1
	Age	0.18	0.06	0.002	0.06, 0.29
	BMI—normoweight	−6.9	2.4	0.003	−11.5, −2.3
	BMI—overweight	−2.1	1.9	0.268	−5.7, 1.6
	BMI—obese	0			
18–45 years	Constant	78.3	3.7	0.000	71.1, 85.5
	BMI—normoweight	−9.44	2.87	0.001	− 3.8, 10.8
	BMI—overweight	−5.1	2.5	0.039	−9.9, −0.26
	BMI—obese	0			
46–80 years	Constant	73.4	3.6	0.000	66.4, 80.4
	Sodium-to-potassium ratio	3.8	1.1	<0.001	1.6, 5.9

CI—confidence interval.

**Table 5:** Dietary intake of salt, sodium, and potassium expressed in g/day. Mean ± SD, [median].

	Sodium Intake (g/Day)	Salt Intake (g/Day)	Potassium Intake (g/Day)	Dietary Na-to-K (mmol) *
Total sample (*n* = 163)	2.29 ± 1.07 [2.05]	5.8 ± 2.7 [5.2]	1.37 ± 0.68 [1.21]	2.9
Normotensive (*n* = 88)	2.32 ± 1.09 [2.22]	5.9 ± 2.8 [5.6]	1.47 ± 0.79 [1.30] ^a^	2.9
Hypertensive (*n* = 75)	2.25 ± 1.06 [1.94]	5.7 ± 2.7 [4.9]	1.24 ± 0.51 [1.17]	3.0
Aged 18–45 years				
Normotensive (*n* = 64)	2.45 ± 1.12 [2.36]	6.2 ± 2.9 [6.0]	1.43 ± 0.73 [1.24]	3.1
Hypertensive (*n* = 20)	2.32 ± 0.94 [2.16]	5.9 ± 2.4 [5.5]	1.19 ± 0.52 [1.03]	3.0
Aged 46–80 years				
Normotensive (*n* = 24)	1.95 ± 0.92 [1.85]	5.0 ± 2.3 [4.7]	1.60 ± 0.94 [1.36] ^b^	2.3
Hypertensive (*n* = 55)	2.22 ± 1.10 [1.91]	5.6 ± 2.8 [4.8]	1.26 ± 0.52 [1.20]	3.0

Significant differences between normotensive and hypertensive: ^a^ *p* = 0.025, ^b^ *p* = 0.039. * ratio: median values.
